# Elastic deformations of bolalipid membranes

**DOI:** 10.1039/c5sm02635k

**Published:** 2016-02-28

**Authors:** Timur R. Galimzyanov, Peter I. Kuzmin, Peter Pohl, Sergey A. Akimov

**Affiliations:** a Laboratory of Bioelectro chemistry, A.N. Frumkin Institute of Physical Chemistry and Electrochemistry, Russian Academy of Sciences, 31/4 Leninskiy Prospekt, Moscow 119071, Russia; b Department of Theoretical Physics and Quantum Technologies, National University of Science and Technology “MISiS”, 4 Leninskiy Prospect, Moscow 119049, Russia; c Institute of Biophysics, Johannes Kepler University Linz, Gruberstrasse 40-42, Linz, 4020, Austria

## Abstract

Archaeal membranes have unique mechanical properties that enable these organisms to survive under extremely aggressive environmental conditions. The so-called bolalipids contribute to this exceptional stability. They have two polar heads joined by two hydrocarbon chains. The two headgroups can face different sides of the membrane (O-shape conformation) or the same side (U-shape conformation). We have developed an elasticity theory for bolalipid membranes and show that the energetic contributions of (i) tilt deformations, (ii) area compression/stretching deformations, (iii) as well as those of Gaussian splay from the two membrane surfaces are additive, while splay deformations yield a cross-term. The presence of a small fraction of U-shaped molecules resulted in spontaneous membrane curvature. We estimated the tilt modulus to be approximately equal to that of membranes in eukaryotic cells. In contrast to conventional lipids, the bolalipid membrane possesses two splay moduli, one of which is estimated to be an order of magnitude larger than that of conventional lipids. The projected values of elastic moduli act to hamper pore formation and to decelerate membrane fusion and fission.

## Introduction

Much of the membrane’s mechanical behavior has been attributed to the protein scaffold that is anchored to the bilayer core.^1^ However, the lipid bilayer dominates the total cellular area compliance and bending stiffness.^2^ Moreover, the lipid bilayer’s mechanical properties are determinants of fusion and fission,^3^and the lipid core’s resistance to tilt and bending makes significant contributions to the energetics of membrane protein conformational transitions.^4^ Transmembrane signal transduction is thought to invoke the assembly of liquid-ordered domains.^5^ In turn, registration of ordered domains from the two monolayers is governed by line tension^6^ and, probably to a minor degree, by the mutual attraction of stiff regions in both monolayers, since their registration minimizes spatial restraints on membrane undulations, *i.e.* maximizes entropy.^7^ The process of phase separation into domains, as well as tether formation, cell shape changes, and budding all require that the lipids in the two monolayers slide against each other. This sliding is dominated by the interleaflet drag that appears to be much larger than the in-layer viscosity.^8^ However, it is totally unclear what governs lipid membrane reshaping in the absence of interleaflet drag. This is the case in archaea’s cell membrane, which is formed by unique components, so-called bolalipids (bipolar lipids), as opposed to membranes of bacteria and eukaryotes. Bolalipids are believed to be responsible for the phenomenal stability of archaeal membranes, *i.e.* they allow archaea to exist under extreme conditions, such as high pressure (~400 atm), high temperatures (~ 100 °C), high methane concentrations and very low or high environmental acidity.^9^ In contrast to “conventional” lipids, bolalipids consist of two polar heads and two hydrocarbon chains. In a hydrophilic environment, these lipids self-assemble into monolayers.^10^ In addition, bolalipid membranes are considered promising materials for various scientific and engineering applications,^11,12^ which emphasizes the necessity of investigating their distinctive mechanical properties.

Theoretical investigations of conventional lipids’ mechanics have been carried out in the framework of microscopic and macroscopic models. Microscopic models are represented by various molecular dynamic models^13^ and analytical solutions of statistical mechanics equations.^14^ Macroscopic models use the elasticity theory to treat membranes as a continuum elastic medium. Here we have focused on the lipid membrane elasticity theories. The first elasticity theory for conventional lipid membranes was developed by Helfrich.^15^ Despite the simplicity of Helfrich’s model, it was successfully utilized for theoretical investigations of membrane structures and membrane-associated phenomena.^16–19^ Another big advance towards complete elasticity theory was work accomplished by Hamm and Kozlov,^20^ in which the authors accounted for the bilayer’s intrinsic structure within the framework of so-called tilt deformation. This theory is still widely used for the investigation of various membrane processes and phenomena, such as poration, fission, fusion, and domain formation.^6,21–29^ These theory-based models enable the systematization of available experimental data and possess substantial predictive power. However’ the afore-mentioned elasticity theory still requires an adaptation for bolalipid membranes.

Bolalipids have been experimentally investigated for a long time.^11^ However, not much theoretical research has been carried out’ and all of it was completed in the framework of microscopic models: by means of molecular dynamics^13,30^ and analytical solutions of equations of statistical mechanics.^14,31^ A macroscopic elasticity theory for bolalipid membranes has not yet been developed.

**Fig. 1:**
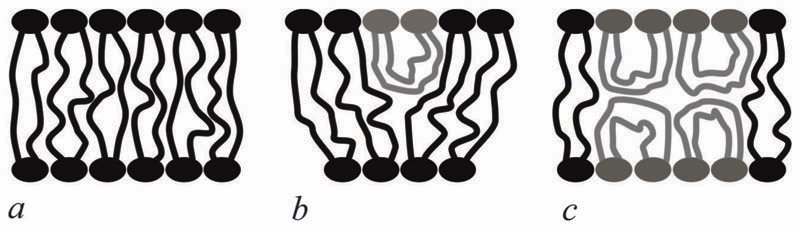
Possible bolalipid configuration in the membrane: (a) O-shape; (b) U-shape and the O-shape mixture; (c) U-shape forming bilayer structure.

**Fig. 2:**
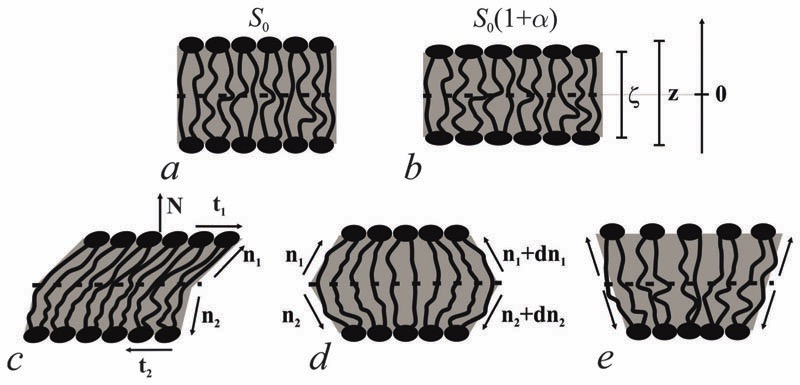
Deformations of bolalipid membranes. (a) Unstrained membrane patch; (b) uniformly stretched patch; (c) uniformly tilted patch; (d) symmetric splay deformations; (e) antisymmetric splay deformations. The bars show the different scales of the ζ and z-axes of the *Oxy*ζ and the *Oxyz* coordinate systems, respectively.

Bolalipids have two conformations: (1) the so-called, O-shapes, in which polar heads are located on different sides of the membrane (Fig. 1a); (2) the so-called U-shapes, in which both polar heads are located on the same side of the membrane (Fig. 1b and c).

Nuclear magnetic resonance experiments revealed that bolalipid membranes contain about 10% U-shapes and 90% O-shapes.^10^ Numerical experiments^13^ predict that the U-shape content depends on the particular experimental setup and may reach up to 60%. A membrane that mainly consists of O-shapes is likely to differ in its mechanical properties from a membrane in which two monolayers interact at the membrane midplane. The elasticity formalism has not yet been developed for the bolalipid membranes. The main aim of the present work is to fill that gap.

Firstly, we derive a general expression for the energy surface density of bolalipid membranes that exclusively consist of O-shaped lipids. As a starting point, we use the general elasticity theory of lipid membranes.^20^ Secondly, we consider U-shapes’ contribution to the elastic energy. Thirdly, we suggest possible experiments and theoretical estimations for defining elasticity moduli and others parameters of the model.

## Statement of the problem

We treat the membrane as a continuous medium which can be subjected to elastic deformations. We aim for the development of an elasticity theory for bolalipid membranes and assume that all deformations are small, so we calculate their energy up to the second order. First, we will only consider bolalipid membranes that consist of O-shapes which thus possess mirror symmetry with respect to the surface that passes through the middle of the membrane in its undeformed state, the so-called “midplane”.

We abide by the previously established algorithm for conventional lipid monolayers.^20^ For convenience, we reproduced the basic equations without excessive mathematical details. Eqn (1) is the general expression for the elastic energy *F* of a laterally liquid medium, written up to the second-order term:^20^

(1) $$\text{d}F=\text{d}V\left[\sigma_{\text{L}}\varepsilon +\frac{1}{2}\lambda_{\text{L}}\varepsilon^{2}+\frac{1}{2}\left(4\lambda_{\text{T}}\right)u_{z\beta }u^{z\beta }\right],$$

where *u_i_* denotes the components of the displacement vector **u** = **r** – **r**_0_ in local tangential Cartesian coordinates *Oxyz*. **r**_0_ and **r** are the radius-vectors of the volume element in the non-deformed state and deformed states, respectively. *u_ij_* is the deformation tensor components: $u_{ij}=\frac{1}{2}\left(\nabla_{i}u_{j}+\nabla_{j}u_{i}\right)+{\left({\left(\nabla_{\text{u}}\right)}^{\text{T}}\nabla_{\text{u}}\right)}_{ji};$ σ_L_, *λ*_T_, and *λ*_L_ are elastic moduli. **u**’s components are related to the lateral expansion ɛ of a volume element by the volumetric incompressibility condition: (1 + ɛ)(1 + Δ*_z_u_z_*) = 1. With the second order terms of *ɛ* it reads: Δ*_z_u_z_* = –*ɛ* + *ɛ* ^2^ +… The deformations are further written in terms of *ɛ* rather than through **u**, for convenience.

The final expression for *F* is written in terms of splay and tilt deformations.^20^ Tilt deformations are characterized by the tilt-vector **t**. It describes the deviation of the average direction **n** (also called “director”, the unit vector) of lipids from the normal **N** to the membrane surface: **t** = **n**/(**nN**) — **N**. Splay deformations are characterized by the mean curvature *J* and the Gaussian curvature *k* of the pivotal surface of lipid monolayers. By definition, a surface is called pivotal when it does not stretch upon splay. Experimental evidence locates the pivotal surface of “normal” phospholipids in the region of the carbonyl groups.^18^ The curvatures are also expressed through **n**: *J* = –div(**n**), $k=\frac{\partial n_{x}}{\partial x}\frac{\partial n_{y}}{\partial y}.$

Bolalipid membranes’ splay deformations (Fig. 2e) would locate the pivotal surface in the vicinity of the membrane midplane. However, for symmetric barrel-like deformations (Fig. 2d), two pivotal surfaces are required as this deformation resembles the symmetric splay of conventional lipid bilayers. They should be located near the head-group regions. It thus appears more convenient to abandon the pivotal surface and to define all deformations with respect to the surface at the membrane midplane. The drawback of such an approach is that we can no longer consider splay and compressing/stretching to be independent of each other.

A vector field of unit normal **N** to the midplane defines the shape of the midplane. Characterizing membrane deformations requires a pair of unit field vectors **n**. Otherwise the membrane would be reduced to an infinitesimally thin film with some independent internal structure, merely defined by the bolalipid’s tilt. Such a description would neither capture highly curved membranes nor the influence of local disturbances, like those displayed upon protein insertion. Moreover, it would fail to describe the case of asymmetric content of U-shape molecules (see Fig. 1b). With a pair of **n**, the average orientation of bolalipids in the upper and the bottom parts of the membrane can be described relative to the midplane (see Fig. 2). All parameters corresponding to the upper and lower membrane halves will be denoted by indices 1 and 2 (see Fig. 2). In the unstrained symmetric membrane, the midplane is flat and **N**, **n**_1_ and **n**_2_ are collinear. Bolalipid membranes are considered both laterally liquid and locally volumetrically incompressible^6,20,24–29^ which are similar to membranes made from conventional lipids.

## Solution to the problem

We introduced an additional local Cartesian coordinate system *Oxyζ*. Its origin *O* coincides with the local tangential Cartesian coordinate system *Oxyz* that has already been introduced. It is located at the midplane. For both systems **N** is directed along the *z* axis and *Oxyz* forms the local tangential basis. However, in the *Oxyz* system, the scale of the *Oz* axis does not change upon deformation of a membrane element; in the *Oxy*ζ system the *Oζ* axis linearly scales with the deformation along this axis (see Fig. 2a and b).

### Tilt deformation of bolalipid membranes

In tilt deformation, both directors deviate from **N** (Fig. 2c). Membrane thickness remains unchanged, since the membrane is volumetrically incompressible (compare Fig. 2a and c).

Tilt can be described by the following dependence of **u** on *z* ^20^:

(2) $$\text{u}=\left\{\begin{array}{ll} {\text{t}}_{1}\cdot z, & z>0,\\ -{\text{t}}_{2}\cdot z, & z<0. \end{array}\right.$$

For small deformations: **t** ≈ **n** — **N**. The only nonzero deformation tensor components *u_z_*_β_ take the following form: $u_{z\beta }=\frac{1}{2}\left[t_{1,\beta }\cdot \theta \left(z\right)+t_{2,\beta }\cdot \theta \left(-z\right)\right]\;\left(\beta =x,y\right),$ where *θ*(z) is the Heaviside step function, defined as *θ(z)* = 0 for *z* < 0 and *θ(z)* = 1 for *z* > 0. By inserting these deformation tensor components into eqn (1) to find the integral over the membrane thickness, we obtained the contribution of tilt deformation to *F*:

(3) $$\begin{array}{rl} \text{d}F_{\text{t}} & =\frac{\lambda_{\text{T}}(z)}{2}\left(t_{1}^{2}\theta (z)+t_{2}^{2}\theta (-z)\right),\\ F_{\text{t}} & =\frac{1}{2}K_{\text{t}}t_{1}^{2}+\frac{1}{2}K_{\text{t}}t_{2}^{2},\\ K_{\text{t}} & =\int_{0}^{h}\lambda_{\text{T}}(z)\text{d}z=\int_{h}^{0}\lambda_{\text{T}}(z)\text{d}z \end{array}$$

where *h* is half of the membrane thickness. Eqn (3) does not contain any cross-terms on tilts *t*_1_ *t*_2_ from the upper and bottom parts. The reason is that the cross-terms would be linked to contributions from the average curvature of the lipid hydrocarbon tails. They are negligible in comparison to *F*, as indicated by the observation of a substantial population of U-shapes in bolalipid membranes.^10,13^ That is, the energy of such a significantly curved hydrocarbon chain is comparable with the characteristic energy of thermal fluctuations, *k*_B_ *T* ~ 4 × 10^−21^ J. Consequently, it is safe to assume that chain bending occurs at a negligible energetic expense.

### Splay and compressing/stretching of bolalipid membranes

The local curvature *J* of a lipid monolayer is given as^21^ *J* = –div(**n**). Splay does not lead to the shearing of volume elements so that *u_z_*_β_ = 0. Splay contributions to F are due to the stretching of the hydrocarbon region (*ε* ≠ 0). Treating a small deformed patch of conventional lipids in terms of a curvilinear trapezium^20^ yields proportionality between *e* and three variables: *J, k,* and α (the relative area change of the whole membrane): *ε* = *α* + *ζJ* + *ζ* ^2^ *k*. *ζ* is the distance between the midplane and the volume element (Fig. 2d and e). α adopts values different from zero if the membrane is subjected to lateral tension, σ. For further calculations, we note that *J* and *k* are of the first and second orders of smallness, respectively.^20^

In contrast, deformations of bolalipid membranes are parameterized by two pairs of curvatures: *J*_1_, *k*_1_ and *J*_2_, *k*_2_. Thus, stretching a volume element located at distance ζ from the midplane takes the following form:

(4) $$\varepsilon =\left(1+\varepsilon_{\text{m}}+\alpha \right)\left(1+\zeta J_{1}+\zeta^{2}k_{1}\right)\theta (\zeta )+\left(1+\varepsilon_{\text{m}}+\alpha \right)\left(1-\zeta J_{2}+\zeta^{2}k_{2}\right)\theta (-\zeta )-1,$$

The equilibrium midplane stretching value, ɛ_m_, upon splay deformation (see Fig. 2d) is found by minimizing *F*.

For further calculations, we switch from the *Oxy*ζ coordinate system to the *Oxyz* coordinate system using the volumetric incompressibility condition for a membrane patch of area *A*_0_: $A_{0}z=A_{0}\int_{0}^{\zeta }\left(1+\varepsilon \left(\zeta^{\prime }\right)\right){\mathrm{d\zeta}}^{\prime }$. *z* may be expressed *via* ζ as:

(5) $$\left\{\begin{array}{ll} z=\left(1+\alpha +\varepsilon_{m}\right)\left(\zeta +\frac{1}{2}J_{1}\zeta^{2}+\frac{1}{3}k_{1}\zeta^{3}\right), & \zeta >0\\ z=\left(1+\alpha +\varepsilon_{m}\right)\left(-\zeta +\frac{1}{2}\zeta^{2}J_{2}+\frac{1}{3}k_{1}\left(-\zeta^{3}\right)\right), & \zeta <0 \end{array}\right.$$

Substituting eqn (5) into eqn (4), we obtain ɛ(z):

(6) $$\varepsilon =\left\{\begin{array}{ll} \alpha +\varepsilon_{m}+J_{1}z-\frac{1}{2}{J_{1}}^{2}z^{2}+k_{1}z^{2}, & z>0,\\ \alpha +\varepsilon_{m}-J_{2}z-\frac{1}{2}{J_{2}}^{2}z^{2}+k_{1}z^{2}, & z<0. \end{array}\right.$$

We obtain the splay contribution to the free energy, *F_j_* by inserting eqn (6) into eqn (1) and zeroing *u_zβ_* (pure splay). Assuming laterally uniform tension allows for the minimization of *F_J_* with respect to ɛ_m_:

(7) $$\varepsilon_{\text{m}}=-\frac{\left(J_{1}-J_{2}\right)}{2K_{\text{A}}}\int_{0}^{h}\lambda_{\text{L}}z\text{d}z$$

(8) $$F_{J}=\frac{1}{4}B_{\text{d}}{\left(J_{1}-J_{2}\right)}^{2}+\frac{1}{4}B_{\text{s}}{\left(J_{1}-J_{2}\right)}^{2}+K_{\text{G}}\left(k_{1}+k_{2}\right)-\tau_{1}J_{1}-\tau_{2}J_{2}+K_{\text{A}}\alpha^{2},$$

where the elastic moduli are defined as follows: $\begin{array}{ccc} B_{\text{d}}=\int_{0}^{h}\left(\lambda_{\text{L}}-\sigma_{\text{L}}\right)z^{2}\text{dz}, & B_{\text{s}}=B_{\text{d}}-\frac{1}{K_{\text{A}}}{\left(\int_{0}^{h}\lambda_{\text{L}}\mathrm{zdz}\right)}^{2}, & K_{\text{G}}=\int_{0}^{h}\sigma_{\text{L}}{\text{z}}^{2}\mathrm{dz}, \end{array}K_{\text{A}}=\int_{0}^{h}\lambda_{\text{L}}\text{dz},\tau_{1}=-\int_{0}^{h}\sigma_{\text{L}}\left|z\right|dz,\tau_{2}=\int_{-h}^{0}\sigma_{\text{L}}\left|z\right|\text{dz}.\tau_{1}=\tau_{2}$ for symmetric membranes. *B*_d_ and *B*_s_ respectively characterize the splay of the whole membrane (the curvatures of the membrane parts above and below the midplane with equal absolute values and opposite signs) and the intrinsic membrane splay that acts to preserve a flat membrane on average (the curvatures of the membrane parts are equal, both in absolute value and sign). *K*_G_ is the Gaussian curvature modulus. With these definitions, *F_J_* of an arbitrarily deformed small patch of a bolalipid membrane can be expressed as:

(9) $$F_{J}=\frac{B_{s}}{4}{\left(J_{1}+J_{2}-2J_{\text{ss}}\right)}^{2}+\frac{B_{\text{d}}}{4}{\left(J_{1}-J_{2}\right)}^{2}+\frac{K_{\text{t}}}{2}\left({t_{1}}^{2}+{t_{2}}^{2}\right)+K_{\text{G}}\left(k_{1}+k_{2}\right)+K_{\text{A}}\alpha^{2},$$

where *J*_ss_ is determined by *B*_s_ *J*_ss_ = τ_1_ = τ_2_. *J*_ss_ is similar to the spontaneous curvatures of conventional lipid membranes.

We have disregarded mixed deformations, such as simultaneously occurring splay and tilt, since they are energetically decoupled as has been derived for conventional lipids. The reasoning is that a linear vector term cannot be part of an energy expression. However, the second-order cross-term of the scalar quantity splay (div(**n**)) and the vector quantity tilt (tilt-vector **t**) does not obey that requirement. Thus, tilt and splay must be considered independently.

In contrast to the expressions for *F* of conventional lipid bilayers, the cross-term for the curvatures of opposing membrane parts exists in the corresponding expressions for bolalipids. The cross-terms for the tilts of opposing membrane parts, for *α* and *J*_1_ or *J*_2_ are absent. That does not transform the midplane surface into a neutral one, since midplane stretching ɛ_m_ still depends on curvature (eqn (7)). It only means that the energy contributions from the deformations induced by the lateral tension s and by the applied torques are independent of each other. The cross-term in *J* originates from the fact that the upper and lower halves share a common midplane. Model accuracy allows us to neglect the Gaussian curvature cross-terms.

### Limiting case

O-shape membranes topologically differ from conventional lipid bilayers. Nevertheless, symmetric deformations of both bolalipid and conventional membranes should be describable *via* the same equations, because their midplanes are similarly deformed. For conventional lipids, the pivotal surface is located in the region of the carbonyl groups.^18^ In our model, the pivotality (inextensibility) of this surface is equivalent to the infinitely large stretching modulus in the carbonyl group region, *λ*_L_(*z = h*) → ∞. We thus obtain $\varepsilon_{m}=-\frac{\left(J_{1}-J_{2}\right)}{2}h,$ which matches the corresponding value in the model for conventional monolayers.^20^ Upon substitution of *λ*_L_(*z* = h) → ∞ eqn (9) reduces to

(10) $$F_{J}=2\frac{1}{2}B_{\text{s}}{\left(J-J_{\text{ss}}\right)}^{2}+2\frac{K_{\text{t}}}{2}t^{2}+2K_{\text{G}}k\;B_{\text{s}}=\sigma_{\text{L}}\int_{o}^{h}z\left(2h-z\right)dz+\lambda_{\text{L}}\int_{o}^{h}{\left(h-z\right)}^{2}dz=\lambda_{\text{L}}\int_{0}^{h}z^{\prime }dz^{\prime }-\sigma_{\text{L}}\int_{0}^{h}{\left({z^{\prime }}^{2}-h^{2}\right)}^{\prime }dz^{\prime }=\int_{o}^{h}\left(\lambda_{\text{L}}-\sigma_{\text{L}}\right){z^{\prime }}^{2}dz^{\prime }\;J_{\text{ss}}=-\frac{1}{B_{\text{s}}}\int_{o}^{h}\sigma_{\text{L}}\text{zdz}$$

In the penultimate equation we substituted *h — z* for *z*′ to match the previously used notations.^20^ We obtained *J*_ss_ by assuming a mechanically stable membrane: $\int_{0}^{h}\sigma_{\text{L}}\left(z\right)\text{dz}=0$. Thus, for defined conditions eqn (10) coincides with the corresponding equations for conventional monolayers.^20^

### Spontaneous curvature

On the one hand, the spontaneous curvature arises in an expression for *F* for a bolalipid layer composed only of O-shape bolalipids (eqn (9)). On the other hand, it is evident that the U-shape lipids would make a stronger contribution to the spontaneous curvature. Combining the contributions of O-shapes and U-shapes is a non-trivial task since bilayers from U-shape molecules do not necessarily obey eqn (9). They are best described by the equations for conventional lipid membranes (eqn (1)). However, if we consider that the concentration of U-shapes is usually below 10%,^10,13^ we can assume that the U-shape concentration is of the same order of smallness as the elastic deformations. Thus, the effect of U-shapes on the elastic moduli must be negligible, since accounting for it would involve third or fourth order corrections to *F*. Under such conditions, the presence of U-shapes affects only those terms that are linear on deformations, *i.e.* *τ*_1_, *τ*_2_. Thus, we may limit all further considerations to the linear terms *x*_1_ and *x*_2_ of U-shape concentrations, since the limited accuracy of the model does not allow us to discern the contribution of higher order terms. Assuming that the headgroups of the U-shapes localize toward the external surface of the bolalipid membrane (Fig. 1b), we must account for different areas *a*_1_ and *a_2_* of the two head-group regions. Repeating the derivation of eqn (8) for asymmetric membranes yields eqn (11) for its parameters:

(11) $$\tau_{1}=-\int_{0}^{h}\sigma_{\text{L}}|z|\text{d}z-\frac{1}{2}\frac{a_{2}-a_{1}}{a_{1}}\int_{0}^{h}\lambda_{\text{L}}|z|\text{d}z+x_{1}\tau_{\text{u}}=\tau_{0}+x_{1}\tau_{\text{u}}-\frac{\tau_{\text{A}}}{2}\frac{a_{2}-a_{1}}{a_{1}},\;\tau_{2}=-\int_{-h}^{0}\sigma_{\text{L}}|z|\text{d}z-\frac{1}{2}\frac{a_{2}-a_{1}}{a_{1}}\int_{0}^{h}\lambda_{\text{L}}|z|\text{d}z+x_{2}\tau_{\text{u}}=\tau_{0}+x_{2}\tau_{\text{u}}+\frac{\tau_{\text{A}}}{2}\frac{a_{2}-a_{1}}{a_{1}},\;\begin{array}{rl} \tau_{\text{A}}= & \int_{0}^{h}\lambda_{\text{L}}|z|\text{d}z=\sqrt{K_{\text{A}}\left(B_{\text{d}}-B_{\text{s}}\right)} \end{array}$$

The coefficient *τ*_u_ reflects the additional stress induced by the presence of U-shapes. *τ*_1_ and *τ*_2_ exclusively act to affect *J*. Since the per surface areas occupied by one U-shape molecule and by one O-shape molecule differ twofold, the ratio (*a*_2_ — *a*_1_)/*a*_1_ can be estimated as (*a*_2_ — *a*_1_)/*a*_1_ ≈ *x*_2_ — *x*_1_. Thus, the effect of up to 10% U-shapes on F_*J*_ of bolalipid membranes may be taken into account by inserting into eqn (9) *J*’s dependence on *x*_1_ and *x*_2_:

(12) $$\begin{array}{rl} F_{J}= & \frac{B_{\text{s}}}{4}{\left(J_{1}+J_{2}-2J_{\text{s}0}-J_{\mathrm{su}}\left(x_{1}+x_{2}\right)\right)}^{2} \end{array}+\frac{B_{\text{d}}}{4}{\left(J_{1}-J_{2}-J_{\mathrm{du}}\left(x_{1}-x_{2}\right)\right)}^{2}+\frac{K_{\text{t}}}{2}\left(t_{1}^{2}+t_{2}^{2}\right)+K_{\text{G}}\left(\kappa_{1}+\kappa_{2}\right)+K_{\text{A}}\alpha^{2}$$

where $J_{s0}=\tau_{0}/B_{s},J_{su}=\tau_{u}/B_{d}+\tau_{A}/B_{d}$. The spontaneous curvatures *J*_su_ and *J*_du_ reflect the contribution of U-shapes. *J*_su_ is an additive to *J*_s0_, the spontaneous curvature of O-shapes. *J*su and *J*_du_ are linear in composition since the concentration of U-shapes is small, and there is no reason to suspect non-ideal mixing (*e.g.* phase separation). Even if nonideal mixing would occur, it is unlikely, that its effect would significantly alter the model prediction, since the model is of limited accuracy.

Eqn (12) ignores the entropic contribution of mixing U-shapes with O-shapes, which should be encountered when the deformational energy is comparable to or smaller than the thermal energy *k*_B_T. Any lateral inhomogeneity of U-shapes may favor membrane deformations that are laterally non-uniform. For this reason we estimate the spontaneous curvature of U-shapes below.

### Elasticity modulus of tilt

The elasticity moduli *B*_s_, *B*_d_, *K*_t_, *K*_G_, *K*_A_ (compare eqn (12)) should be measured experimentally, calculated from microscopic models, or otherwise assessed. Simple calculations show that *K*_t_ of conventional lipids should be close to the surface tension of the oil-water interface,^20^ which was experimentally confirmed.^32^ Extending the same considerations to bolalipid membranes, we estimate its *K*_t_ to be equal to that of conventional lipids, *i.e. K*_t_ ~ 50 dyn cm^–1^.

All other elasticity moduli depend on lipid structures and properties, thereby precluding this type of simple estimation. They should be experimentally measured. However, assessing *K*_G_ is very difficult even in the case of conventional lipids. At the same time, *K*_G_G only needs to be accounted for in a narrow and peculiar set of problems, in which membrane topology changes. Below we focus on how to estimate *B*_s_ and *B*_d_.

1. ***B*_d_**. *K*_a_ and *B*_d_ measurements are commonly based on monitoring the increment in vesicular membrane area upon application of hydrostatic pressure. The change in surface area of a giant unilamellar vesicle (GUV) is associated with undulations and expansion of the area per lipid molecule.^33–36^ GUVs with a diameter of about 10 μm are well suited for this purpose because the average curvature is small. Since *J*_1_ and *J*_2_ have different signs, *J*_1_ + *J*_2_ is much smaller than *J*_1_ — *J*_2_. This means that *B*_d_ and *K*_A_ can be determined in such experiments.^33-36^ The energetic contribution of the Gaussian curvature is constant because the system’s topology does not change during the experiment (Gauss-Bonnet theorem).
2. ***B*_s_**. Luminal conductivity measurements of lipid nanotubes that are pulled from the membrane represent an alternative method for the determination of elastic properties.^25,37–39^ The measured conductivity allows the determination of the inner nanotube radius *R_2_* = 1/*J*_2_. For conventional lipids, *R_2_* depends both on splay modulus and membrane lateral tension.^25,37,38^ *J*_1_ + *J*_2_ cannot be assumed to be small because *R*_2_ is comparable with membrane thickness. Moreover, the U-shaped bolalipids are likely to laterally redistribute. Due to the cylindrical symmetry of the nanotube, tilt deformations do not appear. In addition, Gaussian curvature does not contribute to the energy associated with changes in nanotube radius *R*_2_.

Applying lateral tension s to a cylindrical tube alters *F* as follows:

(13) $$\begin{array}{rl} F= & \frac{2\pi }{J}\left[\frac{1}{4}B_{\text{s}}{\left(J_{1}+J_{2}-J_{\mathrm{ss}}\right)}^{2}+\frac{1}{4}B_{\text{d}}{\left(J_{1}-J_{2}-J_{\mathrm{sd}}\right)}^{2}\right]+\sigma \left(\frac{2\pi }{J_{1}}+\frac{2\pi }{J_{2}}\right) \end{array},$$

where *J*_1_ = (1/*J* + *h*)^−1^, *J*_2_ = −(1/*J* — h)^−1^, *J*_ss_ = 2*J*_s0_+ *J*_su_(*x*_1_ + *x*_2_), *J*_sd_ = *J*_du_(*x*_1_ — *x*_2_). The indices “1” and “2” correspond to external and internal parts of the membrane that form the nanotube, respectively. We define tube radius *R* = 1/J at the membrane midplane; *h* is equal to half of the membrane thickness, *J*_ss_ and *J*_sd_ are spontaneous curvatures (eqn (10)). The energy density is multiplied by the area of the non-deformed state,^40^ which with sufficient accuracy may be assumed to be equal to the area of the nanotube midplane. *F* given by eqn (13) should be minimized with respect to *J* and the concentration of U-shapes, *x*_1_ and *x*_2_. As a result we find *R* as a function of σ. *B*_s_ is then obtained by varying σ *via* the application of transmembrane voltage.^37^

For conventional lipids, the elastic moduli are much greater than the characteristic energy of thermal fluctuations, *k*_B_ *T*. For instance, *B*_s_ is about^36^ 10*k*_B_ *T*. Similarly, we may thus assume that the lateral distribution of U-shapes is only governed by *F*. The formation of nanotubes occurs much faster than the lateral redistribution of membrane components with non-zero spon-taneous curvature^39^ (U-shapes). Consequently the U-shape concentrations in the internal and the external parts of the nanotube membrane immediately after formation are equal to the U-shape concentration in a flat membrane and *J*_sd_ = 0. Minimizing *F* given by eqn (13) with respect to nanotube curvature, we obtain *B*_d_:

(14) $$B_{\text{d}}=\frac{-\frac{{\left(1-(h/R{)}^{2}\right)}^{4}R^{3}}{R^{\prime }}-B_{\text{s}}\left(\left(1+3(h/R{)}^{2}\right)\left(1-(h/R{)}^{2}\right)hJ_{\text{ss}}-6(h/R{)}^{2}\left(1+(h/R{)}^{2}\right)\right)}{3(h/R{)}^{4}+8(h/R{)}^{2}+1}$$

R’ is the derivative of *R* with respect to σ (*R’* < 0 for real systems); *R’* could be measured experimentally. The expression can be simplified for large *R (h/R* ≪ 1):

(15) $$B_{\text{d}}\approx -\frac{R^{3}}{R^{\prime }}-B_{\text{s}}hJ_{\mathrm{ss}}$$

The nanotube radius subsequently relaxes due to the lateral redistribution of U-shapes in both monolayers. The relaxation is governed by the independent minimization of elastic energy in each monolayer. As a result, a local effective transversal asymmetry of U-shaped molecules may emerge. The characteristic time amounts to about 1 s for conventional lipids (dioleoyl-phosphatidylethanolamine, DOPE).^39^ The resulting equilibrium curvature of the nanotube as well as *x*_1_ and *x*_2_ can be obtained by minimizing *F* (eqn (13)) with respect to *R*, *x*_1_, and *x*_2_. Energy minimization demands the absence of U-shapes (possessing positive *J*_s_) on the internal part of the nanotube having negative curvature. This yields the expression for *B*_d_ in the limit of large *R*:

(16) $$B_{\text{d}}\approx -\frac{R^{3}}{R^{\prime }}+B_{\text{s}}$$

Thus, *B*_d_ can be obtained from experiments with GUVs, while *B*_s_ and *J*_ss_ are attainable by measuring nanotube radii in and out of equilibrium.

## Discussion

We have obtained a general expression for the surface energy density of elastic deformations for bolalipid membranes which consist of two types of molecules: O-shapes and U-shapes. *F* includes cross-terms for (i) curvatures of opposing membrane parts and (ii) curvatures with U-shape concentrations. Tilt cross-terms are absent because they are determined by the average bending of hydrocarbon chains which make a negligibly small contribution to *F*. For specific cases where transmembrane peptides are embedded in a bilayer, the absence of the tilt cross-term would no longer apply. In the framework of our model, however, this should be taken into account by means of coupling boundary conditions set on deformations rather than the free energy functional.

Our theoretical considerations should help to ascertain the major differences in the roles archaeal and conventional lipids play during cellular processes that involve membrane reshaping. Archaea possess at least three distinct membrane remodelling systems.^41^ The first uses an archaeal actin-related protein, the “cell division A” CdvA protein. The second is comprised of the bacterial-type system FtsZ. The third alternative cell division apparatus is homologous to the eukaryotic ESCRT-III (endosomal sorting complex required for transport). Remarkably enough, membrane scission by the yeast ESCRT-III complex does not require a special type of lipid in addition to certain amounts of anionic lipids to preserve a negative net charge^42^—a requirement that can easily be met by bolalipids. However, the energetics of scission should be fundamentally different when bolalipids are involved because their elastic moduli are different. In contrast to scission, early stages of fusion, *i.e.* hemifusion and fusion pore formation depend on lipid curvature,^43^ and it would be interesting to see how bolalipids may meet these requirements. To get a first impression about the energetics involved in membrane remodelling, we will estimate the basic elastic parameters of bolalipids from our theory.

Estimates of *B*_s_, *B*_d_, and *J*_s_ are attainable from simple considerations: symmetric splay of bolalipids is analogous to the symmetric splay of conventional lipid bilayers. Comparing the energetic costs for their splay $E_{c}=2\frac{BJ^{2}}{2}$ with eqn (10) indicates that *B*_s_ is of the same order of magnitude as that of conventional lipids. In contrast, the case of antisymmetric splay, (*J*_1_ = —*J*_2_), could not have been reduced to splay-like deformations of a conventional lipid bilayer, since in that case, the dividing surfaces are necessarily subjected to compression/ stretching deformations. The stretching, ε_*J*_, is approximately equal to ε_*J*_ ≈ *h*(*J*_1_ — *J*_2_)/2 = *hJ*_1_. By assuming that curvature-like deformations dominate the energetic costs, and that their contribution is similar to that of compression/stretching deformations of conventional lipid membranes, the total deformational energy in the antisymmetric case adopts the form: $E\approx 2\frac{K_{\text{A}}\alpha^{2}}{2}+2\frac{BJ_{1}^{2}}{2}=\left(K_{\text{A}}h^{2}+B\right)J_{1}^{2},$ where *K*_A_ = 120 mN m^−1^ = 30*k*_B_ *T* nm^−2^ for most types of conventional lipids. Comparing *E* with *F* in eqn (9) enables the assessment of *B*_d_ as: *B*_d_ = *K*_A_ *h* ^2^ + *B* ≈ 130*k*_B_ *T*, which is an order of magnitude larger than the splay modulus of conventional lipid membranes.

**Fig. 3:**
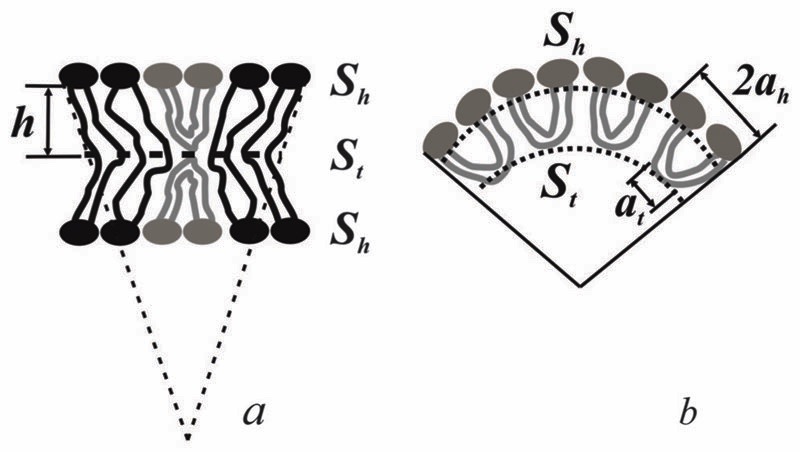
(a) U-shaped lipids induce the spontaneous curvature in bolalipid membranes. (b) Toy-model of a bolalipid monolayer from U-shapes. *a*_h_ is the area per lipid headgroup, *a*_t_ is the area at the membrane midplane, *h* is half of the thickness of the bolalipid membrane

The spontaneous curvature of a monolayer composed of U-shapes can be estimated using a toy-model. Symmetrical insertion of U-shaped bolalipid molecules into a membrane that consists of O-shapes (Fig. 3a) enlarges the membrane surface, *S*_h_, more than it increases the midplane area, *S*_t_. In the limiting case of a pure U-shape monolayer, the spontaneous curvature is positive, since all polar headgroups are located at the same side of the membrane (Fig. 3a and b).

If *a*_h_ is the area per lipid headgroup of both O-shapes and U-shapes, and if *a*_t_ is the area of an O-shaped or U-shaped molecule at the membrane midplane, we attain the following expressions for the membrane surface areas:

(17) $$\begin{array}{rl} S_{\text{h}}/N & =(1-x)a_{\text{h}}+x\cdot 2a_{\text{h}}=(1-x)a_{\text{h}}\\ S\text{t}/N & =(1-x)a_{\text{t}}+xa_{\text{t}}=a_{\text{t}}\\ J_{1} & =J_{2}\approx \frac{1}{h_{0}}\frac{S_{\text{h}}-S_{\text{t}}}{S_{\text{t}}}=\frac{1}{h_{0}}\frac{(1-x)a_{\text{h}}-a_{\text{t}}}{a_{\text{t}}} \end{array}\;$$

where *h*_0_ is the equilibrium thickness of the monolayer from U-shapes, which can be taken as equal to *h*—half of the thickness of membranes made from O-shapes; *N* is the number of total lipid molecules. eqn (17) assumes linear dependence of the headgroup and tail region areas *S*_h_ and *S*_t_ on *x*. *J*_1,2_ can be found as:

(18) $$\begin{array}{rl} J_{1} & =J_{2}\approx \frac{1}{h}\frac{S_{\text{h}}-S_{\text{t}}}{S_{\text{t}}}=\frac{1}{h}\frac{(1+x)a_{\text{h}}-a_{\text{t}}}{a_{\text{t}}} \end{array}$$

Since *J*_1,2_ = 0 for a layer of O-shapes, 2a_h_ must be approximately equal to *a*_t_. Consequently, eqn (17) transforms into:$J_{1}=J_{2}\approx \frac{1}{h}x.$ According to eqn (12) this corresponds to $J_{s0}=0,J_{su}\approx \frac{1}{h}.$. The spontaneous curvature, *J*_du_, can be found as:

(19) $$\begin{array}{rl} J_{\mathrm{du}}= & \tau_{\text{u}}/B_{\text{d}}+\tau_{\text{A}}/B_{\text{d}}=\frac{J_{\mathrm{su}}B_{\text{s}}+\sqrt{K_{\text{A}}\left(B_{\text{d}}-B_{\text{s}}\right)}}{B_{\text{d}}} \end{array}\approx \frac{B_{\text{s}}/h+K_{\text{A}}h}{K_{\text{A}}h^{2}}\approx \frac{1}{h}\approx J_{\mathrm{su}}$$

Eqn (18) and (19) allow the estimation of the energy, *E*_ves_, that is stored in large closed vesicles with symmetrical lipid composition. In the case of bolalipids, Eves is determined by *B*_d_. *E*_ves_ is independent of vesicle radius R: $E_{ves}\approx 4\pi R^{2}\cdot 1/4B_{d}{\left(4/R\right)}^{2}=16\pi B_{d}\approx 6500k_{B}T$. For vesicles made from conventional lipids, Eves does also not depend on radius, but with only 500*k*_B_ *T* it is tenfold smaller. However, *E*_ves_ of bolalipid vesicles decreases significantly when more U-shapes face the outer surface than the vesicle lumen. To account for lipid asymmetry, we fix *R* and minimize *F_J_* (eqn 12) with respect to the U-shape concentrations in both membrane halves, *x*_1_ and *x*_2_. This allows us to obtain the equilibrium concentrations *x*_1_ = 4*h*/*R*, *x*_2_ = 0, which in turn yield $E_{ves}\approx 4\pi R^{2}\cdot 4B_{s}R^{2}=16\pi B_{s}\approx 500k_{B}T$. Thus lipid asymmetry brings *E*_ves_ down to the value obtained for conventional lipids. However, the reduction of *E*_ves_ comes at a substantial cost: for vesicles with an R of 40 or 100 nm, U-shapes have to be enriched in the outer half by *x*_1_ = 20% or *x*_1_ = 8%, respectively.

Based on the estimates for *E*_ves_, we expect that the fusion of bolalipid membranes requires an asymmetrical U-shape distribution. Components with non-zero spontaneous curvature substantially alter the rate of membrane fusion even in the case of conventional lipid membranes.^3,44^ It decelerates if the contacting (proximal) leaflets have positive spontaneous curvature, and accelerates if the positive spontaneous curvature is acquired by the distal monolayers. Thus, enrichment of U-shapes in the distal halves of the membrane should facilitate membrane fusion. The asymmetry has to be locally restricted to the fusion zone. While lysolipids, which play that role in conventional membranes, may be produced at little cost by phospholipases and selectively enriched by protein imposed curvature, the corresponding mechanisms in bolalipid membranes are not known. Both translocation of a charged bolalipid head-group and protein-induced bending of the bolalipid membrane are certainly energetically much more costly than in the case of conventional lipid bilayers.

We conclude that bolalipids’ unique chemical structure sustains the unique stability of archaeal membranes. Their self-assembly into a monolayer, instead of into a bilayer as is the case with conventional membranes, should significantly hinder membrane reshaping by fusion and fission. From our estimations, bolalipids possess a splay modulus that is an order of magnitude larger than that of conventional mammalian lipids. It acts to further hamper fusion and inhibit pore formation, thus allowing archaea to maintain the membrane barrier to ions and other molecules even in extremely aggressive environments. However, the projected price for this stability is rate deceleration in cell division or endocytotic uptake.
